# Thermal Stability of Dexamethasone—Evaluation with Regard to Modern Medicinal and Pharmaceutical 3D-Printing Applications

**DOI:** 10.3390/molecules30214234

**Published:** 2025-10-30

**Authors:** Roman Svoboda, Roman Vrbenský, Jan Honzíček, Mária Chromčíková

**Affiliations:** 1 Department of Physical Chemistry, Faculty of Chemical Technology, University of Pardubice, Studentská 573, 532 10 Pardubice, Czech Republic; 2 Institute of Chemistry and Technology of Macromolecular Materials, Faculty of Chemical Technology, University of Pardubice, Studentská 573, 532 10 Pardubice, Czech Republic; 3 FunGlass, Alexander Dubček University of Trenčín, Študentská 2, 911 50 Trenčín, Slovakia; 4 VILA—Joined Glass Centre of the IIC SAS, TnUAD, FChPT STU, Študentská 2, 911 50 Trenčín, Slovakia

**Keywords:** dexamethasone, thermogravimetry, thermal decomposition, kinetic predictions

## Abstract

The high-temperature thermal stability of dexamethasone (DEX) was systematically investigated under nitrogen and air atmospheres using non-isothermal thermogravimetry at heating rates of 0.1–20 °C·min^−1^. The thermal decomposition was found to initiate below the melting temperature, proceeding via a three-step pathway that generated a complex mixture of volatile and condensed by-products (~10% solid residuum at 550 °C). Kinetic modeling was realized using the single-curve multivariate kinetic analysis (sc-MKA), and was based on the autocatalytic framework with temperature-dependent parameters, combined with consequent reaction mechanisms. An excellent agreement of the theoretical model with the experimental data enabled reliable predictive extrapolations to pharmaceutical processing conditions. Whereas the onset of degradation was observed at ~180–190 °C, significant decomposition rates (>1% mass loss during first 5 min) were only reached above 220 °C, well above the processing windows of most pharmaceutical polymers. Consequently, dexamethasone can be considered thermally stable for hot-melt extrusion and fused deposition modeling, except in high-temperature-processing applications involving polymers such as, e.g., polylactic acid, polyvinyl alcohol, or thermoplastic polyurethanes. Importantly, the study highlights that reliable kinetic predictions require measurements across a broad heating-rate range and in both oxidizing and inert atmospheres, with special emphasis on low heating rates (≤0.2 °C·min^−1^), which proved critical for capturing early-stage degradation. These findings provide a rigorous kinetic framework for ensuring safe incorporation of DEX into advanced pharmaceutical and medical device formulations.

## 1. Introduction

**Scheme 1:**
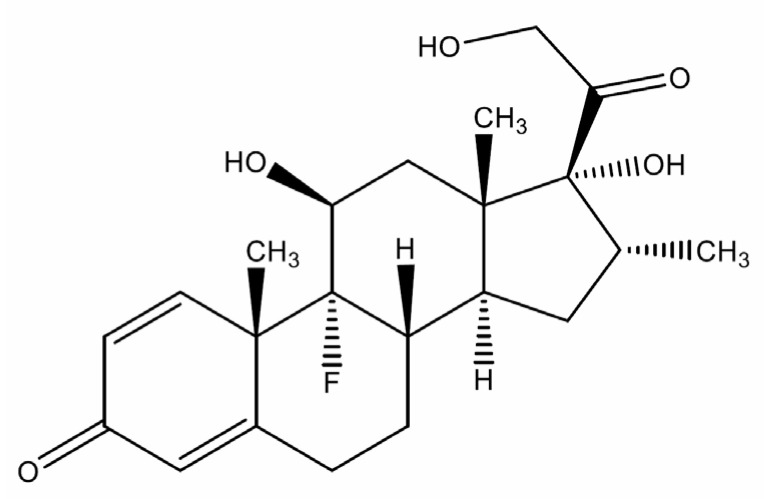
Chemical structure of dexamethasone.

Dexamethasone (DEX; see Scheme 1 for its chemical structure) is a potent synthetic glucocorticoid with high anti-inflammatory and immunosuppressive efficacy, low mineralocorticoid activity, and favorable pharmacokinetics. Mechanistically, it exerts its therapeutic effect by binding to the glucocorticoid receptor, altering gene transcription, and thereby suppressing pro-inflammatory cytokines and upregulating anti-inflammatory mediators [1]. Clinically, dexamethasone is widely prescribed in numerous conditions: management of acute and chronic asthma and chronic obstructive pulmonary disease exacerbations, autoimmune and rheumatologic disorders such as rheumatoid arthritis and systemic lupus erythematosus, and hematologic malignancies, including leukemia, lymphoma, and multiple myeloma, where it is used both for its cytotoxic synergy and its palliative benefits [2,3]. In neurology, DEX is the drug of choice to mitigate cerebral edema due to trauma or tumors [4], and in ophthalmology, it is used topically, intraocularly, or as intravitreal implants for uveitis and macular edema [5,6]. It is also given adjunctively in meningitis to reduce neurologic sequelae [7]. More recently, dexamethasone was shown in the landmark RECOVERY trial to reduce mortality in patients with COVID-19-associated acute respiratory distress syndrome, which further reinforced its clinical significance [8]. Its versatility extends to perioperative settings for the prevention of postoperative nausea and vomiting, as well as to veterinary medicine [9]. The broad therapeutic spectrum, favorable dose–response characteristics, and inclusion in the World Health Organization Model List of Essential Medicines highlight dexamethasone’s global importance [10].

Nowadays, the landscape of pharmaceutical technology is shifting toward patient-tailored and multifunctional drug delivery systems, enabled by advanced processing technologies such as hot-melt extrusion (HME) and additive manufacturing (3D printing). HME is a solvent-free, continuous process in which an active pharmaceutical ingredient (API) is intimately mixed with a polymeric carrier and optional plasticizers under elevated temperature and shear, forming amorphous solid dispersions or controlled-release matrices [11,12]. The technique is now widely adopted in the pharmaceutical industry for improving solubility, controlling release, and fabricating new dosage forms [13]. Coupling HME with fused deposition modelling (FDM) 3D printing has revolutionized personalized medicine by enabling the fabrication of complex geometries, patient-specific doses, and multi-drug constructs [14,15]. Drug-loaded filaments can be prepared via extrusion and directly printed into tablets, orodispersible films, implants, and scaffolds with customized release kinetics [16,17]. The versatility of polymers used—ranging from polyvinyl alcohol (PVA), polyethylene oxide (PEO), hydroxypropyl methylcellulose (HPMC), Eudragit, Soluplus, and ethylcellulose—provides further opportunities for tailoring mechanical and release properties [18,19]. Recent studies demonstrate successful applications such as theophylline [20], aripiprazole [21], indomethacin [22], and ibuprofen [23] incorporated into printable filaments, showing that extrusion and printing can be scaled from proof-of-concept to industrial production. Despite these advances, the thermal stability of the API during HME/FDM remains a central challenge.

In addition to oral dosage forms, many innovative delivery systems exploit polymers, coatings, and biodegradable materials for local release of dexamethasone or other therapeutic molecules. For example, dexamethasone-loaded biodegradable implants have been developed to prevent pericardial adhesions, achieving localized drug release and reduced systemic toxicity [24]. Implant-mediated drug delivery systems in canine mandibles achieved prolonged plasma exposure compared with intramuscular administration [25]. Hydrogels combined with porous microspheres have been engineered to provide sustained release of DEX while promoting bone regeneration [26]. Likewise, poly(lactic-co-glycolic acid) (PLGA) microparticles fabricated via electrospraying have been used for co-delivery of dexamethasone and ropivacaine, prolonging analgesic effects after surgery [27]. In ocular drug delivery, thermoresponsive in situ gels and micelle-based systems have been developed to extend ocular residence and reduce dosing frequency [28]. Orthopedic and dental applications include metallic implants coated with dexamethasone to reduce local inflammation and improve osseointegration [29]. Drug-releasing sutures represent another important class, where antibiotics, anti-inflammatories, or growth factors can be incorporated via coatings, melt-spinning, or embedding within fiber matrices to promote wound healing and reduce infection rates [30,31]. These examples demonstrate the scale of opportunities for incorporating dexamethasone into novel drug–device combinations.

A key consideration across all of these technologies is that HME, 3D printing, and many implant/suture fabrication methods involve elevated thermal and shear conditions. HME typically requires processing temperatures in the 80–160 °C range, while FDM printing nozzles often operate between 150 and 220 °C, depending on the polymer [32,33]. Such exposure may trigger degradation pathways in thermally labile APIs, compromising drug potency, generating toxic by-products, and altering drug release performance. Dexamethasone, in particular, is known to undergo chemical transformations under thermal and oxidative stress [34]. Thus, rigorous investigation of its thermal degradation kinetics is crucial to define safe processing windows, select compatible polymer carriers, and design formulations that maintain stability during manufacturing.

Despite the widespread therapeutic applications of dexamethasone and the increasing adoption of HME and 3D printing in pharmaceutical manufacturing, significant knowledge gaps remain regarding its behavior under processing-relevant conditions. Most available studies address dexamethasone stability in aqueous solutions, under photolysis, or in biological environments, but systematic solid-state kinetic investigations under thermal stress are scarce [35,36,37]. Furthermore, the influence of formulation variables such as the processing atmosphere (air vs. inert) on degradation kinetics has not been fully elucidated. Without this information, it is challenging to define safe processing windows, optimize formulation design, or predict long-term stability of dexamethasone-containing drug–device products. With this motivation, the present study will be focused on an investigation of the high-temperature decomposition kinetics under both air and inert atmospheres. The state-of-the-art physico-chemical modeling will be used to make predictions of the thermal decomposition behavior under conditions relevant for HME and FDM, and to assess the suitability of DEX for the contemporary pharmaceutical and medical formulations/products.

## 2. Results

The present section will be split into two parts—in the first part, the thermogravimetry (TGA), differential thermal analysis (DTA), spectral, and microscopic results will be introduced; in the second part, the DEX decomposition kinetics will be presented.

### 2.1. Raw Data

**Figure 1:**
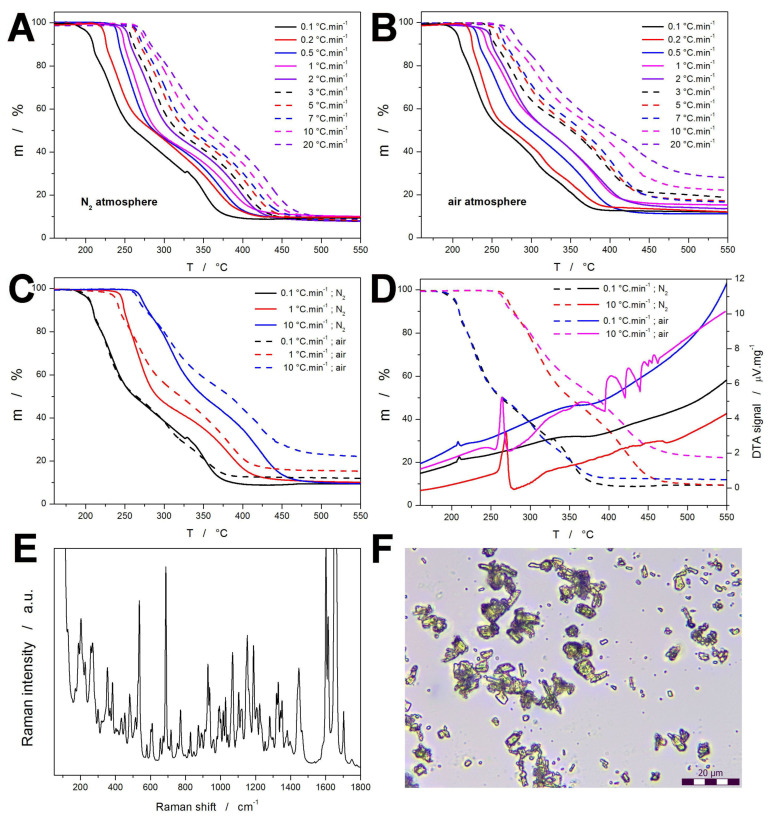
(**A**) Set of TGA curves measured at different *q^+^* under N_2_ atmosphere. (**B**) Set of TGA curves measured at different *q^+^* under air atmosphere. (**C**) Comparison of selected TGA curves measured under both atmospheres. (**D**) Comparison of selected TGA and DTA curves; exothermic effects evolve in the downward direction. (**E**) Raman spectrum of as-purchased DEX. (**F**) Optical micrograph of as-purchased DEX powder.

The thermogravimetric data of the DEX decomposition measured at a series of different heating rates (*q^+^*) in the N_2_ and synthetic air atmosphere are shown in Figure 1A,B. Starting with the measurements performed under N_2_, the true onsets of the initial mass losses occur between 183 and 264 °C for the *q^+^* span of 0.1–20 °C·min^−1^; for the endsets, the corresponding temperature range is 410–505 °C. The mass loss appears to be split into three consequent stages: 100% → 87–89%; 87–89% → 41–47%; and 41–47% → 8–10%. The solid residuum had a tar-like character. The temperature shift of the TGA decomposition curves follows the expected trend, i.e., the mass loss signal shifts to higher temperature (*T*) with increasing *q^+^*. This trend (mathematically explained in Section 2.2) is given by the increasingly shorter time provided for the decomposition reaction at higher *q^+^* paired with the *T*-dependent rate constant (generally increasing with ↑ *T*). In the case of the DEX decomposition proceeding in the air atmosphere (Figure 1B), the onsets and endsets occur in the following respective ranges: 188–260 °C and 397–537 °C. Whereas the former is practically similar for both atmospheres, the endset temperature exhibits a significantly broader *T* span when measured in the air atmosphere. This can be explained by the specific chemical influence of O_2_, which on one hand promotes earlier decomposition of labile reaction sites (through joint fragmentation/oxidation reactions), and on the other hand partially consumes/gasifies char and tar products (prolonging the mass loss response). The decomposition under the air atmosphere exhibits three steps (similar to that in N_2_). However, the corresponding mass losses are (at the second and third steps) significantly different: 100% → 87–90%; 87–90% → 54–61%; and 54–61% → 11–28%. This indicates that the only partial access of O_2_ (most probably the consequence of slow diffusion of air through the degraded material layers at high *q^+^*) leads to the formation of a larger portion of the unideally burnt products (char and tar) that gasify at significantly higher *T*.

Despite the multistep mass loss of DEX clearly indicating that the thermally induced fragmentation of the DEX molecule is gradual and complex (resulting in a variety of different products/by-products), even through an extensive literature review, no study reporting on the thermal decomposition products of DEX was found. Based on the general decomposition patterns found for various corticosteroids, the following attribution can be suggested for the three main (TGA-identified) decomposition steps.

In the first step (260–290 °C at 10 °C·min^−1^; ~15% mass loss), one can expect dehydration (loss of H_2_O from hydroxyl groups), cleavage of weak side-chain bonds (e.g., C17 side chain), partial defluorination (generation of HF), release of small carbonyls, aldehydes and ketones (formaldehyde, acetaldehyde, and acetone), or even hydrocarbons (CH_4_, CO + CO_2_). In the second decomposition step (290–340 °C at 10 °C·min^−1^; ~35% mass loss), major scission of the side chains and cleavage of the DEX D ring (junctions between rings C-D) are expected with the following products: larger oxygenated fragments (acetylates, C4–C8 aldehydes and ketones, and short-chain substituted furans), substituted aromatics (benzene, toluene, phenol, and cresols), steroid mid-molecular-weight fragments (species characterized in mass spectrometry with *m*/*z* = 319–393), and furan and furfural derivatives originating from rearrangement of oxygenated ring fragments. The third TGA mass loss step (340–360 °C at 10 °C·min^−1^; ~40% mass loss) is likely to represent a full ring breakdown, aromatization, condensation, and charring, producing, e.g., CO and CO_2_, H_2_, CH_4_, HF, ketenes, benzenediols, and polycyclic aromatics (naphthalene, anthracene, etc.) [38,39,40,41,42,43,44,45,46,47]. In the light of these estimated attributions, the increased residual mass observed for the decomposition in air at high *q^+^* may be associated with a rapid release of oxygenated intermediates (aldehydes, furans, phenols, and mid-MW steroid fragments), which outpaces their volatilization and combustion. Instead, these reactive species may undergo secondary polymerization and oxidative crosslinking, producing condensed aromatic structures that accumulate as carbonaceous char. Whereas oxygen generally promotes radical coupling, under rapid heating, it does not have sufficient time to fully combust the residue [38,39,40,41,42,43,44,45,46,47].

The direct comparison of the TGA curves selected for the two atmospheres (shown in Figure 1C) indicates that the differences in decomposition behavior increasingly manifest with increasing *q^+^*, leaving the measurements performed at *q^+^* = 0.1 °C·min^−1^ practically identical until the last step initiates. The TGA curves cross during the second decomposition step, which evidently makes this step significantly slower during the access of O_2_, determining the main source of the discrepancy (as suggested above). The complementary DTA curves are shown in Figure 1D, where the main feature is the endothermic melting peak of DEX. For pure DEX, the onset of the melting peak is reported [48] to be at *T_m_* = 262–264 °C, which is in agreement with our measurements performed at 10 °C·min^−1^. In contrast, at low *q^+^*, the melting peak is shifted to a much lower *T* (~206–209 °C), which is a clear consequence of the decomposition process already altering the DEX structure, causing the *T_m_* to decrease. Interestingly, even this melting process of a significantly degraded DEX manifests itself as a single and relatively sharp peak. This indicates that the initial decomposition products are most probably fully gaseous, not producing the liquefaction, through either a eutectic (double peak) or impurity-influencing (peak with prolonged, slow onset) mechanism. It is also worth noting that the heat changes associated with the actual decomposition are not very pronounced, showing only several exothermic effects during the decomposition in air at 10 °C·min^−1^. This indicates that at low *q^+^*, the oxidation has no pronounced effect, i.e., the primary decomposition products are not subject to a major secondary oxidation. At high *q^+^*, the decomposition mechanism changes (as suggested above), and the oxidative crosslinking probably takes place, producing the series of peaks. Their periodical/interruptive nature is probably due to the macroscopic diffusion through the droplets of the molten and partially decomposed matter.

In Figure 1E,F, the supplementary measurements by Raman and optical microscopies are presented for the as-purchased crystalline DEX powder. The DEX Raman spectrum corresponds well to the earlier reported data [49,50,51,52] with the following assignments of the main bands: 3200–3400 cm^−1^ (stretching O–H vibration) corresponds to hydroxyl groups; 3010–3080 cm^−1^ (stretching =C–H vibration) arises from olefinic protons of the unsaturated steroid backbone; strong aliphatic modes appear at 2850–2980 cm^−1^ (stretching C–H vibration of CH_3_ and CH_2_ groups); the carbonyl groups give a band at 1670–1750 cm^−1^ (stretching C=O vibration); a conjugated double bond system contributes at 1600–1640 cm^−1^ (stretching C=C vibration); the 1460–1510 cm^−1^ band corresponds to the bending CH_2_/CH_3_ and skeletal stretching C–C vibrations; the 1300–1375 cm^−1^ band indicates deformation of CH_3_ and mixed CH bending; the band at 1150–1250 cm^−1^ indicates stretching C–O and C–C with CH in-plane bending; the 1000–1100 cm^−1^ band corresponds to the skeletal vibrations including C–O and modes influenced by the C–F substituent; bands at 900–980 cm^−1^ indicate the out-of-plane CH bending and C–C skeletal motions; the 600–800 cm^−1^ region includes the ring deformations and C–F bending; and below 400 cm^−1^, bands are assigned to lattice modes and collective torsions characteristic of solid-state packing. The micro-morphology photographed in Figure 1F then just indicates the average particle size of the DEX powder, which was determined to be ~1–3 μm for the smallest single-grain fraction, and 5–9 μm for the aggregates/agglomerates.

**Figure 2:**
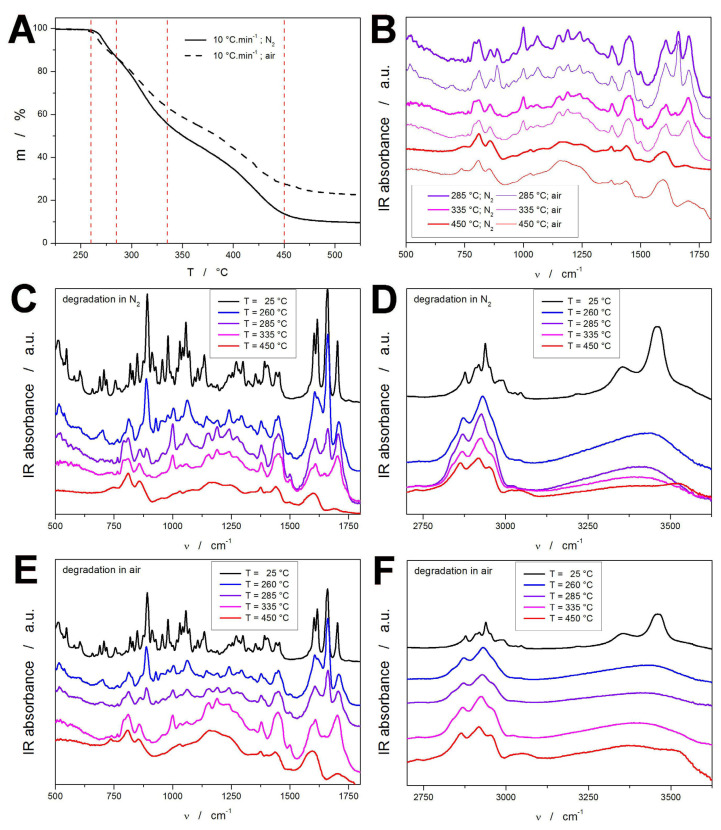
(**A**) Indication of temperatures, to which the FTIR samples were heated. (**B**) Comparison of FTIR spectra obtained under both atmospheres and higher *T*. (**C**–**F**) FTIR spectra obtained in low- and high-wavenumber regions.

To gain further insight into the decomposition of the DEX molecule (apart from the literature information cited above), the FTIR spectroscopy was used to investigate the molecular structure of the samples subject to thermal decomposition during the TGA experiments. For this reason, several characteristic stages of the experiment performed at 10 °C·min^−1^ were selected: 260, 285, 335, and 450 °C, where the first temperature corresponds to the initial few percent of the sample being degraded, and the other three temperatures indicate the final stages of the three TGA mass loss steps (see Figure 2A). It is worth noting that although the measurements were stopped after reaching the selected temperatures and the TGA furnace was immediately opened, the thermal inertia (originating from the heat accumulated in the sample) certainly led to the continuation of the thermal degradation for a few more tens of seconds. The IR spectra depicted in Figure 2 show that already the heating to ~260 °C largely results in significant changes in the DEX structure, merging many bands as a result of the melting and initial bond ruptures (the solid product has already shown a light-tar-like appearance). Particularly striking are the disappearance of the 606 cm^−1^ band (indicating the initial disruption/opening of the steroid ring system, probably starting at the weakest bonds or strained positions), and the appearance of the 1004 cm^−1^ band (which indicates formation of new C–O or C–C bonds, possibly ether-like or oxidized structures initiating from the hydroxyl oxidation) [39,42,43,44,50,51,52].

At 285 °C (after the first decomposition step), the disappearance of the 700 cm^−1^ band and the decrease in the 887 cm^−1^ band indicate changes in the specific cyclic or aliphatic C-H environments, consistent with the further ring opening; the appearance of the 792 cm^−1^ band may correspond to the formation of new C=C bonds, corresponding to the formation of new alkenes via dehydrogenation or dehydration; the occurrence of the 1189 cm^−1^ band suggests the formation of new oxygenated species (esters, peroxides, or enols); the decrease in the 1661 cm^−1^ band then indicates the destruction of the enone structural motif (C=O-C=C conjugation). At 335 °C (the finalization of the second decomposition step), the disappearance of the 887 and 1061 cm^−1^ bands corresponds to the loss of the C–H bending alkene vibrations and C–O stretching vibrations, indicating the decomposition of the unsaturated and alcoholic/ether functional groups. In addition, the disappearance of the 1661 cm^−1^ band signalizes a complete breakdown of the enone system associated with the DEX A ring. This likely means disintegration of the entire steroid skeleton, with the resulting structure consisting of smaller oxygenated fragments (aldehydes, ketones, and carboxylic acids). At 450 °C (the end of the final monitored decomposition step), further disappearance of the 792, 1004, 1189, and 1702 cm^−1^ bands indicates continuing loss of the C–O, C=O, and C=C vibrations, indicating the destruction of the organic structures, with the majority of the solid residuum consisting of carbonaceous material (char) in the case of the decomposition in air, and hydrocarbons (tar) in the case of the decomposition in N_2_ [39,42,43,44,50,51,52]. This difference was also observed visually, after inspecting the solid residue from the 450 °C experiment.

### 2.2. Decomposition Kinetics

To model the decomposition kinetics monitored by the TGA, the standard solid-state kinetic equation [53] is as follows:

(1) $$\frac{d\alpha }{dt}=A\cdot e^{-E/RT}\cdot f(\alpha )$$

where *dα*·*dt*^−1^ is the rate of conversion (with *α* being the degree of conversion scaled between zero and one), *A* is the pre-exponential constant, *E* is the activation energy of the decomposition process, *R* is the universal gas constant (8.314 J·mol^−1^·K^−1^), and *f*(*α*) is a function defining the shape/asymmetry of the decomposition step as a dependency of *α.* The degree of conversion is related to the mass loss data via the following:

(2) $$\alpha =\frac{m_{0}-m}{m_{0}}$$

where *m* is the actual sample mass, and *m*_0_ is the original sample mass. Maximum flexibility of the mathematical description was achieved by using the autocatalytic Šesták-Berggren (*AC*) model function [54]:

(3) $${f(\alpha )}_{AC}=\alpha^{M}{\left(1-\alpha \right)}^{N}$$

where *M* and *N* are the kinetic exponents of the *AC* model. Note that the physico-chemical interpretation of the *M* and *N* values was recently introduced in [55]. Since the present mass loss data exhibit complex kinetic behavior, consisting of three consequent sub-processes (the decomposition of organic molecules belongs inherently among consequent reaction schemes), the actual mathematical framework had to reflect this fact—the following combination of the kinetic and balance equations was used:

(4) $$\frac{dm_{A}}{dt}={-A}_{1}\cdot exp\left(-\frac{E_{1}}{RT}\right)\cdot m_{B}^{M_{1}}\cdot {\left(1-m_{B}\right)}^{N_{1}}$$

(5) $$\frac{dm_{B}}{dt}=A_{1}\cdot exp\left(-\frac{E_{1}}{RT}\right)\cdot m_{B}^{M_{1}}\cdot {\left(1-m_{B}\right)}^{N_{1}}-A_{2}\cdot exp\left(-\frac{E_{2}}{RT}\right)\cdot m_{C}^{M_{2}}\cdot {\left(1-m_{C}\right)}^{N_{2}}$$

(6) $$\frac{dm_{C}}{dt}=A_{2}\cdot exp\left(-\frac{E_{2}}{RT}\right)\cdot m_{C}^{M_{2}}\cdot {\left(1-m_{C}\right)}^{N_{2}}-A_{3}\cdot exp\left(-\frac{E_{3}}{RT}\right)\cdot m_{D}^{M_{3}}\cdot {\left(1-m_{D}\right)}^{N_{3}}$$

(7) $$\frac{dm_{D}}{dt}=A_{3}\cdot exp\left(-\frac{E_{3}}{RT}\right)\cdot m_{D}^{M_{3}}\cdot {\left(1-m_{D}\right)}^{N_{3}}$$

(8) $$m_{D}=1-m_{A}-m_{B}-m_{C}$$

(9) $$m=m\left(t=0\right)+∆m\cdot m_{rel}\left(t\right)$$

(10) $$m_{rel}=w_{1}\cdot \left(1-m_{A}\right)+w_{2}\cdot \left(m_{C}+m_{D}\right)+\left(1-w_{1}-w_{2}\right)\cdot m_{D}$$

where *m_x_* is the normalized mass of the given phase (corresponding to the respective α_x_); the indices *A*–*D* denote the individual states within the following reaction scheme

(11) A→B→C→D

and the coefficients *w_x_* are related to the proportional representation of the individual mass loss steps.

**Figure 3:**
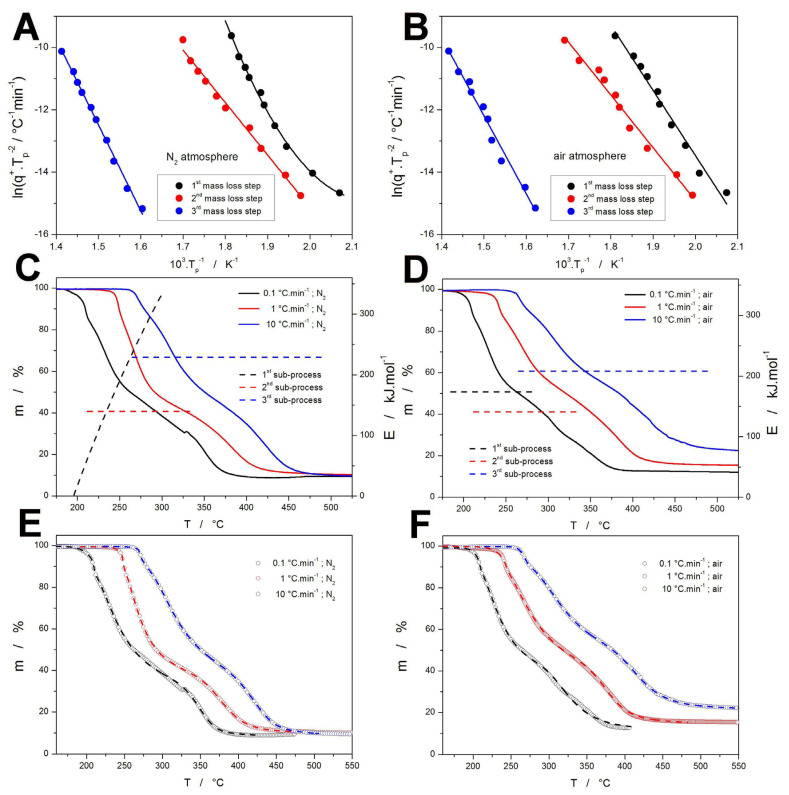
(**A**,**B**) Kissinger plots for the TGA data measured in both atmospheres; points show experimental data, lines correspond to either linear or second-order polynomial fits. (**C**,**D**) Selected TGA curves (left axes and solid lines) and activation energies (right axes and dashed lines) determined from the Kissinger dependencies for the respective sub-processes manifesting in temperature ranges given by the dashed lines. (**E**,**F**) Examples of the TGA curves (points) fit by the kinetic model represented by Equations (4)–(11) (dashed lines) (black: 0.1 °C·min^−1^; N_2_; red: 1 °C·min^−1^; N_2_; blue: 10 °C·min^−1^; N_2_).

With such a complex set of mathematical equations (as described above), the nonlinear optimization is the standard and most reliable solving approach. In order to reduce the number of variables for the optimization, it is customary to independently determine the values of activation energy, *E_x_*. In this regard, the Kissinger equation [56] is the most reliable and robust method [57,58,59]:

(12) $$ln\left(\frac{q^{+}}{T_{p}^{2}}\right)=-\frac{E}{RT_{p}}+const.$$

where *T_p_* is the temperature corresponding to the maximum of the decomposition peak (the point of inflection in the case of the integral kinetic form, such as the *m*-*T* dependencies shown in Figure 1A,B). To determine the *T_p_* values from the mass loss data, d*m*·d*T*^−1^-*T* dependencies were calculated by derivation over Δ*T* = 2 °C. The obtained *T_p_* values were for the three prominent mass loss steps used to construct the Kissinger plots depicted in Figure 3A,B for the two sets of mass loss data (corresponding to the two series of measurements conducted in the N_2_ and air atmospheres). Whereas most of the obtained Kissinger dependencies exhibit acceptable linearity, the dependency measured for the initial mass loss (the first mass loss step) under the N_2_ atmosphere shows a significant deviation in linearity at the lowest *q^+^* (in the low-*T* region). As the linear dependencies provide a single value of *E_x_* (determined according to Equation (12), the curved Kissinger dependencies need to be expressed in terms of the temperature-dependent activation energy *E_x_*(*T*). In the present case, the given Kissinger dependency was fit by the second-order polynomial function, the derivation of which was used to calculate the *E*-*T* dependency. The *E_x_*(*T*) values are for the three sub-processes and the two atmospheres shown in Figure 3C,D together with the selected TGA curves—the dashed lines indicate not only the corresponding *E_x_* but also the approximate temperature range, in which the given sub-process manifests itself within the measured range of *q^+^* (0.1–20 °C·min^−1^).

With the knowledge of *E_x_*(*T*), the nonlinear optimization of Equations (4)–(10) can be used in order to determine the rest of the kinetic parameters. In particular, the single-curve multivariate kinetic analysis (sc-MKA) method [60] can be advantageously used by fixating *E_x_* at the corresponding values for each sub-process (calculating a constant *E_x_*(*T_p_*) value in the case of the first mass loss measured in N_2_), and optimizing each mass loss curve separately. Note that with the independently obtained *E_x_* values, the rest of the kinetic parameters are unambiguously determined [60] for each single measurement, providing the *q^+^*/*T*-dependent description of the decomposition kinetics. The mathematical framework for the nonlinear optimization was based on the determination of the least sum of the squared residue:

(13) $$RSS=\sum_{{First}_{j}}^{{Last}_{j}}{\left({Yexp}_{j,k}-{Ycal}_{j,k}\right)}^{2}$$

where *RSS* is the sum of squared residue, *j* is an index of the given measurement, *First_j_* is the index of the first point of the given curve, *Last_j_* is the index of the last point of the given curve, *Yexp_j,k_* is the experimental value of the point *k* of curve *j*, and *Ycal_j,k_* is the calculated value of the point *k* of curve *j* (calculated in accordance with Equations (4)–(11)). The optimization was conducted using the Levenberg–Marquardt algorithm. The quality of the corresponding mathematical fits is shown in Figure 3E,F. The only deviations (slight) between the experimental and calculated data were identified for the third (final) mass loss steps measured at 0.1 and 0.2 °C·min^−1^. Otherwise, a practically perfect description of the decomposition kinetics was obtained.

**Figure 4:**
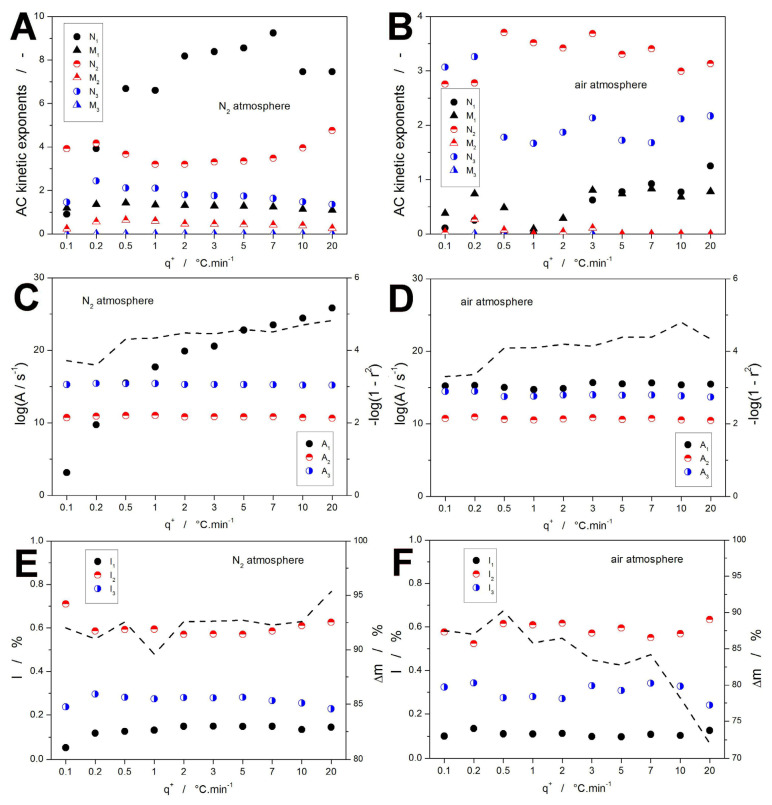
(**A**,**B**) AC kinetic exponents determined by the sc-MKA method for the TGA data measured under both atmospheres. (**C**,**D**) Values of the pre-exponential factor *A* (left axes and points) and correlation coefficients (right axes and dashed lines) obtained from the sc-MKA method. (**E**,**F**) Proportional representation of individual sub-processes (left axes and points) and overall mass loss (right axes and dashed lines).

The sc-MKA results obtained for the TGA decomposition of DEX in the N_2_ and air atmospheres are visualized in Figure 4 and listed in Supplementary Materials. The main benefit of the sc-MKA method is the identification of trends in the *q^+^*/*T*-dependent kinetics. These trends play a major role in the consequent predictions of the kinetic behavior, which are usually calculated based on certain extrapolation from (or conscious selection of) a specific set of kinetic parameters. Often, drug behavior at lower temperatures is of interest in pharmaceutical practice (mainly because the direct experimental monitoring of its behavior would be either extremely time-demanding or below the instrumental detection limits). Here, the most suitable extrapolation is that conducted from the kinetic parameters measured at the lowest *q^+^*, which best imitate the low-*T*-induced slow changes in the API molecular structure. In this view, the present sc-MKA results will be interpreted. Starting with the kinetic exponents *M* and *N* (Figure 4A,B), the generally high *N* values indicate that the corresponding sub-processes are initiated with a rapidly increasing conversion rate, while the autoretardation takes place during the later stages of the process. Mechanistically, such behavior may be associated with a barrier layer being formed from the initial decomposition products (possibly tar-like solid residue), which slows down the release of the gaseous products and/or diffusion of oxygen to the reaction interface. Kinetic predictions-wise, significant changes in the otherwise rather constant trends occur at the lowest *q^+^* (0.1 and 0.2 °C·min^−1^). On the contrary, the pre-exponential factor *A* (Figure 4C,D) does not show similar changes associated with the lowest *q^+^*. Instead, it copies the trends in *E_x_*, as is usual due to the impact of the kinetic compensation effect [61]. These graphs also show the generally very high values of correlation coefficients, closely corresponding to the high quality of the sc-MKA fits already suggested in Figure 3E,F. In Figure 4E,F, the proportional representations of the individual mass loss steps (together with the overall mass loss Δ*m*) are shown—the notation is as follows: *I*_1_ = *m_B_*·100%; *I*_2_ = *m_C_*·100%; and *I*_3_ = *m_D_*·100%. No significant trends (beyond the span of experimental errors) in *I_x_* can be identified. The only notable trend is the decrease in Δ*m* at high *q^+^* in an air atmosphere (as already commented on by Figure 1B).

## 3. Discussion

The present section will be divided into two sub-sections. In the first part, the kinetic predictions of the DEX thermal decomposition will be introduced, based on the kinetic description of the present experimental data (as presented in Section 2). The focus will be on the accuracy of these predictions in relation to the selected set of kinetic parameters, from which the prediction is calculated. In the second part, the present results will be commented on with regard to the potential utilization of DEX in different pharmaceutical polymer matrices processed via HME and FDM.

### 3.1. Kinetic Predictions

The kinetic description of the DEX thermal decomposition (as described in Section 2.2) was achieved by means of the sc-MKA method, which allows for the identification of the kinetic trends associated with changing *q^+^* (or, alternatively, changing temperature ranges during which the chemical reactions proceed). Consequently, this gives the opportunity (and simultaneously a burden of forcing a decision) to choose the kinetic basis (a set of kinetic parameters) for the calculation of the kinetic predictions. In the present section, the differences in the kinetic predictions based on arbitrarily selected sets of initial kinetic parameters (*E_x_*, *A_x_*, *M_x_*, *N_x_*, and *w_x_*) will be introduced, and the impact of the above-mentioned choice will be discussed with regard to the procedures of pharmaceutical HME and FDM. Note that the main point of the predictions is to extrapolate the observed behavior towards otherwise immeasurable conditions (usually low temperatures leading to extremely long reaction times).

In the case of the HME process, the decomposition in air appears to be more relevant, since the reactants (API, matrix polymer, and possible other excipients) are introduced into the extruder in powdered/granulated form, which enables good access of oxygen to the mixture during its heating. Temperature range-wise, in the case of one of the pharmaceutically most relevant polymers, hydroxypropylmethylcellulose (HPMC), the extrusion conditions range between 120 and 170 °C [62]. On the other hand, the FDM process may, from a large part, proceed without major access of O_2_ to the APIs incorporated into the compact (HME-produced) filament. Hence, the N_2_-based decomposition should be more relevant. Note that the key aspect is the oxygen diffusion into the polymer matrix, which is relatively low for the pharmaceutical polymers. The temperature range for FDM is significantly higher, in the case of HPMC, usually ranging between 180 and 210 °C [62], but the exposure to the elevated temperatures is much shorter compared to HME (30–60 s vs. 3–15 min).

**Figure 5:**
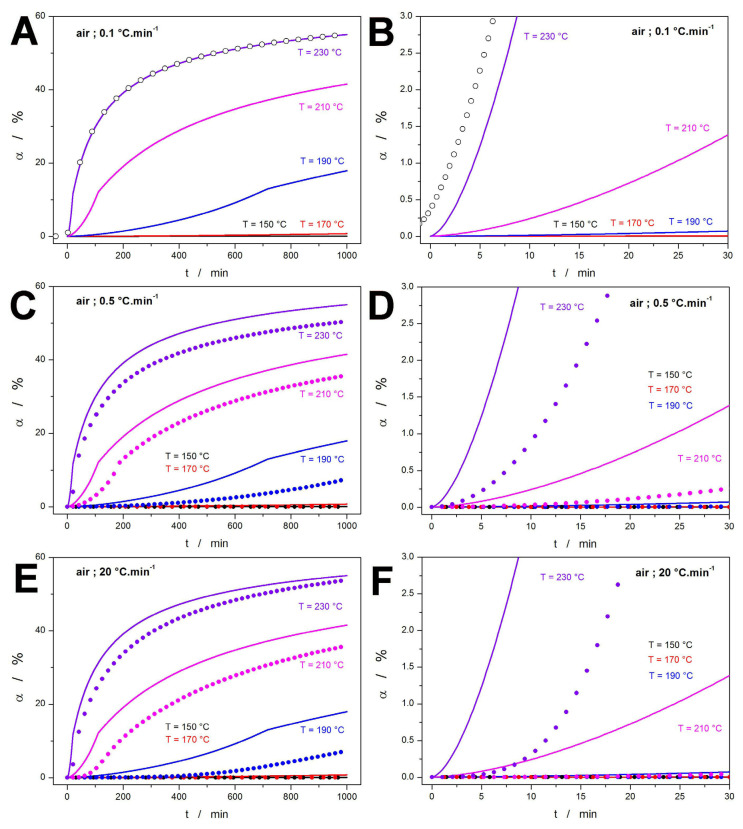
(**A**) Kinetic predictions calculated for selected isothermal annealings (indicated by *T*) using the set of sc-MKA-determined parameters from the measurement performed at 0.1 °C·min^−1^ in air; points depict a prediction for annealing at *T* = 230 °C with preceding heating from 30 °C at 5 °C·min^−1^. (**B**) Zoomed-in graph. (**A**,**C**) Predictions calculated from parameters corresponding to *q^+^* = 0.5 °C·min^−1^ measurement in air (points) compared with the 0.1 °C·min^−1^ predictions from graph (**A**) (lines). (**D**) Zoomed-in graph. (**C**,**E**) Predictions calculated from parameters corresponding to *q^+^* = 20 °C·min^−1^ measurement in air (points) compared with the 0.1 °C·min^−1^ predictions from graph (**A**) (lines). (**F**) Zoomed-in graph (**E**).

Starting with the kinetic predictions based on the TGA measurements in an air atmosphere, the summarizing visualization is given in Figure 5. For the purpose of comparison, the predictions calculated from the parameters obtained for the measurement at 0.1 °C·min^−1^ will be taken as a baseline behavior. The corresponding predictions of isothermal annealing at selected temperatures (150, 170, 190, 210, and 230 °C) are shown in Figure 5A (the version zoomed-in on the initial minutes is depicted in Figure 5B). In addition to the simple simulation of isothermal annealing (initiated directly at the selected annealing temperature), an additional simulation was performed, involving also the preceding heating step at 5 °C·min^−1^ (depicted as points in the two graphs). Clearly, even during the most pronounced exposition to high *T* (heating up to 230 °C before the start of the annealing), the preliminary heating step does not significantly accelerate the consequent decomposition, and its impact can be neglected. From the practical point of view, the relevant (impactful) temperatures for the DEX thermal decomposition with regard to HME and FDM are *T* ≥ 210 °C. However, since these temperatures are normally achieved/needed only for FDM (which lasts several tens of seconds), even the worst-case scenarios (FDM at 230 °C) would result in the degradation from ~0.1%.

The comparison between the predictions based on 0.1 °C·min^−1^ data (lines) and predictions calculated for the kinetic description of the TGA curves measured at 0.5 and 20 °C·min^−1^ (points) is shown in Figure 5C–F. The main goal of this comparison is to consider the justification of the more costly/time-demanding measurements performed at the lowest *q^+^* (0.1 and 0.2 °C·min^−1^). As is apparent, the overall/general kinetic response (depicted in Figure 5C,E) is rather similar, with the errors usually ranging between 5 and 10%, which is perfectly acceptable for the purpose of estimating the rate of the API decomposition. However, if looking at the initial decomposition stages (which are of true interest when the formation of potentially dangerous decomposition products is considered), an order of magnitude difference is observed, with the predictions based on the 0.1 °C·min^−1^ kinetics estimating a much higher (more than ×10) degree of conversion achieved in the first tens of seconds, and minutes. In other words, if only the measurements at *q^+^* = 0.5–20 °C·min^−1^ were performed, an unrealistically low amount of the potentially hazardous decomposition products would be predicted, jeopardizing the patient’s health.

**Figure 6:**
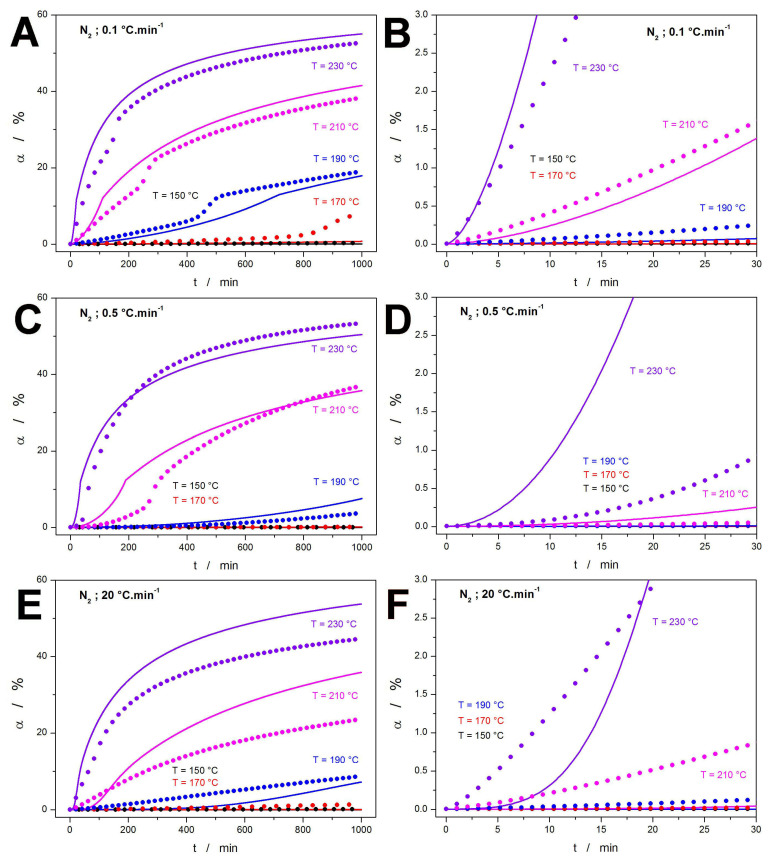
(**A**) Kinetic predictions calculated for selected isothermal annealings (indicated by *T*) using the set of sc-MKA-determined parameters from the measurement performed at 0.1 °C·min^−1^ in N_2_ (points) compared with the 0.1 °C·min^−1^ air-data-based predictions (lines). (**B**) Zoomed-in graph. (**A**,**C**) Predictions calculated from parameters corresponding to *q^+^* = 0.5 °C·min^−1^ measurement in N_2_ (points) compared with the 0.5 °C·min^−1^ air-data-based predictions (lines). (**D**) Zoomed-in graph. (**C**,**E**) Predictions calculated from parameters corresponding to *q^+^* = 20 °C·min^−1^ measurement in N_2_ (points) compared with the 20 °C·min^−1^ air-data-based predictions (lines). (**F**) Zoomed-in graph (**E**).

A similar depiction is shown for the TGA decomposition under a N_2_ atmosphere, shown in Figure 6. Here, the predictions based on the corresponding measurements in O_2_ are used as the baseline behavior (represented by lines). Whereas the overall differences (depicted in Figure 6A,C,E) between the N_2_-based and air-based predictions are not very significant, major deviations can again be found at the initial decomposition stages (Figure 6B,D,F). Interestingly, due to the *E_x_*-*T* dependency being implemented in the kinetic description of the TGA data measured under N_2_, the corresponding predictions do not exhibit a monotonous trend in comparison to their air-based counterparts. Instead, the low-*t* behavior is rather unpredictable using common sense without actually performing the calculations. This further stresses the importance of detailed kinetic measurements under both types of atmospheres and under a broad range of experimental conditions. The latter is also underscored by the comparison of the N_2_ predictions based on different *q^+^* (comparing, e.g., the violet dotted dependencies from Figure 6B,D,F), which again demonstrate the large (otherwise unpredictable) difference associated with the behavior identified at the lowest *q^+^* (0.1 and 0.2 °C·min^−1^). From a practical point of view, considering the HME and FDM in conjunction with HPMC, the N_2_ kinetic predictions do not bring anything new or important. Also, the N_2_ data, which may be more relevant specifically for FDM, show that only at the extreme temperatures (~230 °C), the formation of potentially toxic DEX decomposition products should be considered.

### 3.2. Practical Implications of Kinetic Predictions

The kinetic predictions of the thermal decomposition of DEX (as presented in Section 3.1) show that the critical temperatures, when the decomposition rate would be comparable to processing time-scales (characteristic for the pharmaceutical HME and FDM procedures), are at *T* ≥ 220 °C. This is already mainly the domain of FDM, as the HME process generally does not require full melting of the polymeric matrix, and can proceed at much higher viscosities than the FDM, where the fast nature of the printing process requires smoother flow (ensured by low viscosity) and better rheological properties. In Section 3.1, the usage of DEX in the HME/FDM-processed polymer matrices was considered for the example of HPMC. This is a polymer with rather moderate processing temperatures; hence, the consequent (present) effort to map the processing temperature windows of the most common pharmaceutical polymers. The list is given in the Supplementary Materials, and includes the glass transition and melting temperatures, processing window, and general use of the polymer/excipient. The list shows that the absolute majority of the pharmaceutical polymers have processing temperature windows below 200 °C, where the decomposition of DEX is negligible even at prolonged times, significantly exceeding even the longest HME residence times (~15 min). The most common exceptions (polymers with the commonly utilized processing temperatures ≥200 °C) are as follows: cellulose acetate (used for coatings of enteric drug delivery systems), polylactic acid (production of biodegradable implants), polyvinyl alcohol (water-soluble matrix for orally introduced films), and thermoplastic polyurethanes (implants and drug reservoirs). Since DEX is already used in medical implants to reduce inflammation and fibrosis, its long-term release from the high-*T* processed polymer-based pharmaceutical and medical devices/products is indeed relevant. The incorporation of DEX into PLA, PVA, and TPU was demonstrated in [63,64], [65], and [66,67,68], respectively. All three polymers are compatible with DEX, although the hydrophilic nature of PVA suggests only a limited solubility of DEX. Since for these three polymers the upper limit for the FDM processing is 220–230 °C, the DEX decomposition can proceed to ~0.2–1% of conversion. However, even such a low degree of decomposition can already lead to the formation of potentially hazardous and toxic by-products, such as formaldehyde, HF, or acrolein. It is thus highly recommended to consider the plasticization of these polymer matrices (to lower their melting temperatures), when admixing with DEX during the pharmaceutical FDM.

## 4. Materials and Methods

The crystalline dexamethasone powder was purchased from Sigma-Aldrich (Burlington, MA, USA) in (certified) 99.7% purity. It was stored in a dry, dark environment at 10 °C; it was used in the as-purchased state for all measurements. The qualitative decomposition test was performed by heating up a batch of 100 mg in a glass vial in an oil bath; before the sample started to melt, white smoke had already occurred, indicating a strong decomposition directly from the solid state. Based on this finding, the thermal decomposition of DEX was considered to be the key factor for its processability via the HME and FDM procedures. The quantitative monitoring of the temperature-induced mass loss of DEX was performed by using the STA (TGA) 449 F5 Jupiter instrument (Netzsch, Selb, Germany) equipped with a DSC/TG holder. The STA measurements were performed as a series of heating scans between 30 and 550 °C at *q^+^* = 0.1, 0.2, 0.5, 1, 2, 3, 5, 7, 10, and 20 °C·min^−1^, with each heating scan performed for a fresh sample. It is worth noting here that since the thermal decomposition starts slightly below the melting temperature *T_m_*, the actual sample form matters (contrary to the majority of low-molecular organic compounds, which decompose from the melt). Hence, each powder sample was carefully spread on the bottom of an open Al pan to ensure similar spatial conditions for the heat transfer, contact with atmosphere (either 4.0 grade N_2_ or synthetic air, i.e., 21% of O_2_; 50 mL·min^−1^ in both cases), and potential powder sintering. The raw sample masses varied between 4 and 6 mg. The samples were dried under the flow of N_2_ at 30 °C for 15 min before the measurements were started to remove the adsorbed H_2_O. The supplementary heat flow information provided by the DSC sensor was also monitored. The enthalpy signal calibration was conducted based on the melting peak of DEX. Reproduction of the selected TGA measurements has shown the reproducibility to be ±2 °C along the *T* axis, and ±3% in the relative magnitude of the individual mass loss steps. The accuracy of the determination of the overall mass loss was better than ±2%.

In addition to the main bulk of experimental data provided by the STA instrument, optical microscopy and Raman spectroscopy were used to perform a base characterization of the as-purchased DEX powder. The optical microscope iScope PLMi (Euromex, Duiven, The Netherlands) equipped with a Moticam camera was used in reflection mode to determine the average particle size of the DEX powder. The Raman microscope DXR2 (Nicolet, Thermo Fisher Scientific, Waltham, MA, USA) equipped with the 785 nm excitation diode laser (30 mW, laser spot size 1.6 μm) and CCD detector was used to collect to spectrum for the as-purchased powder to confirm its identity. The spectrum was collected under the following conditions: 20 mW laser power on the sample, 3s duration of a single scan, and 60 scans summed in one spectrum. Infrared spectra were collected on a Nicolet iS50 FTIR spectrometer (Thermo Fisher Scientific) in the region of 4000–400 cm^−1^ (data spacing = 0.5 cm^−1^) using a built-in diamond ATR crystal; 8 scans were averaged in one spectrum.

## 5. Conclusions

The thermal decomposition of DEX in the N_2_ and air atmospheres was studied using non-isothermal thermogravimetry in a broad range of applied heating rates (0.1–20 °C·min^−1^). The TGA analysis revealed that the degradation starts from the solid state (initiating below *T_m_*), producing a complex set of volatile and condensed by-products. The three-step decomposition pathway was described in terms of the consequent reactions based on the autocatalytic model with implemented temperature-dependent kinetics. The kinetic modeling performed by means of the sc-MKA method demonstrated very good agreement with the experimental data, enabling reliable extrapolations to pharmaceutical processing conditions (times and temperatures characteristic for the HME and FDM procedures). The results show that while the degradation onset occurs at ~180–190 °C, significant decomposition rates are only reached above 220 °C, which largely exceeds the typical processing windows of most pharmaceutically relevant polymers—probably only the DEX-containing medical devices (such as implants and stents) based on, e.g., PLA, TPU, and PVA would have to consider the risk of the formation of toxic degradation products during the HME and FDM processing.

On the other hand, the detailed analysis of the kinetic predictions based on different kinetic datasets led to the conclusion that to achieve a complete and comprehensive overview of the thermal degradation process, measurements at a very broad range of *q^+^* and in both oxidizing and non-oxidizing atmospheres need to be performed. The crucial importance of the kinetic measurements performed at *q^+^* ≤ 0.2 °C·min^−1^ (which are not commonly performed) needs to be emphasized. In addition, only the methodically rigorous kinetic analysis respecting the *q^+^*/*T*-dependent trends appears to be suitable for the accuracy requirements associated with the utilization in pharmaceutical practice, where toxic life-endangering products can form during the degradation.

## Data Availability

The original data are available at https://doi.org/10.6084/m9.figshare.30281845.v1.

## Supplementary Materials

The following supporting information can be downloaded at https://www.mdpi.com/article/10.3390/molecules30214234/s1: Table S1: List of common pharmaceutically relevant polymers. Table S2: sc-MKA kinetic parameters obtained for TGA data.
